# Reuse of a Previously Transplanted Heart

**DOI:** 10.1155/crit/5193591

**Published:** 2026-08-07

**Authors:** Alexis Krin, Myriam Addi, Jean Porterie

**Affiliations:** ^1^ Department of Anesthesia and Intensive Care, University Hospital of Toulouse, Toulouse, France, chu-toulouse.fr; ^2^ University Toulouse 3-Paul Sabatier, Toulouse, France; ^3^ Department of Cardiac Surgery, University Hospital of Toulouse, Toulouse, France, chu-toulouse.fr

**Keywords:** cardiac graft reuse, heart transplantation, organ shortage

## Abstract

Heart transplantation remains the gold standard treatment for end‐stage heart failure. However, the persistent shortage of donor organs continues to limit the number of procedures performed worldwide. Several strategies have been developed to expand the donor pool, including the reuse of a previously transplanted cardiac graft, which represents a rare alternative. We report the first case of cardiac graft reuse performed in France, and the ninth case described worldwide. A 61‐year‐old man with end‐stage dilated cardiomyopathy and cardiogenic shock underwent urgent heart transplantation using a graft that had been previously transplanted into another recipient, who subsequently developed brain death. Pretransplant evaluation confirmed preserved graft function, allowing for retransplantation. The postoperative course was initially complicated by vasoplegic shock but evolved favorably. At the 3‐year follow‐up, the patient remained in a good functional status with preserved cardiac function.

**Key Clinical Message**

Reuse of a previously transplanted cardiac graft is an extremely rare but feasible strategy to expand the donor pool. Careful graft assessment and appropriate recipient selection may allow for successful retransplantation with favorable mid‐term outcomes.

## 1. Introduction

Currently, the number of patients awaiting heart transplantation continues to rise, whereas the availability of donor hearts is declining, largely due to a persistent shortage of suitable organ donors. To address this growing disparity and expand the donor pool, several innovative strategies have been developed, including donation after circulatory death, ex vivo cardiac perfusion, and domino‐transplantation procedures. Since 1993, eight cases of retransplantation have been reported worldwide. In this report, we describe a new reuse of a cardiac graft with a 3‐year follow‐up, the first reported in France, and briefly review the literature.

## 2. Case Report

A 61‐year‐old man with end‐stage heart failure secondary to dilated cardiomyopathy was followed at our institution. His blood group was O Rh‐negative. Relevant comorbidities included arterial hypertension and Type 2 noninsulin‐dependent diabetes mellitus. Despite optimal guideline‐directed medical therapy, his clinical condition progressively deteriorated, ultimately leading to hospitalization for atrial fibrillation‐induced cardiogenic shock. Immediate treatment was initiated with antiarrhythmic medication and external electrical cardioversion, which successfully restored the sinus rhythm, in association with norepinephrine infusion. Cardiac evaluation revealed cardiac biventricular insufficiency with an estimated ejection fraction of 20%, and altered right ventricular function. Intravenous Levosimendan was initiated 1 day after hospital admission at a dose of 0.2 *μ*g/kg/min for 48 h. Weaning from norepinephrine was impossible. Pretransplant immunological evaluation revealed no detectable anti–HLA Class I or II antibodies. Right heart catheterization demonstrated a pulmonary vascular resistance of 2.7 Wood units and a mean pulmonary artery pressure of 29 mmHg, confirming eligibility for transplantation (Table 1).

**Table 1:** Clinical timeline of cardiac allograft reuse and recipient outcomes.

Day 0	First heart transplantation
	Total ischemic time: 246 min
**Recipient A**	
POD 1	Extubation
POD 8	Hemorrhagic stroke
	Deep Sylvian hematoma
Brain Death recipient A	Preserved graft function
	No inotropic support
Cardiac allograft reuse	ABO compatible
	Virtual and physical crossmatch negative
	DSA negative
**Recipient B**	**Second heart transplantation**
Day 0	Total ischemic time: 240 min
	Early postoperative period:
	‐ Severe Right ventricular failure ➔ inhaled nitric oxide + Milrinone + Isoproterenol
	‐ Vasoplegic shock ➔ Resolved after vasopressin
POD 3 to POD 20	POD 3 : Extubation
	POD 10 : ICU discharge
	POD 20 : Hospital discharge
**1 Month**	• Preserved graft function
	• No rejection
**3 Months**	• Mild cellular rejection (1R)
**2- and 3-year follow-up**	• NYHA I
	• Excellent quality of life
	• Return to sports activities
	• LVEF 60%
	• No transplant‐related complications

Heart transplantation was proposed 1 day after being listed for transplantation, and the initial donor was a 40‐year‐old man who was declared brain‐dead 1 day after a hemorrhagic stroke. The donor′s blood group was O Rh‐positive, with a medical history of tobacco use and occasional cannabis consumption. Cardiac evaluation in sinus rhythm demonstrated preserved left ventricular systolic function with an ejection fraction of 65% in the absence of inotropic support. The interventricular septal thickness was 8 mm, and no mitral or aortic valvular abnormalities were identified. Coronary angiography was not performed in this case. Low‐dose norepinephrine (0.2 *μ*g/kg/min) was administered for hemodynamic support. The peak high‐sensitivity troponin level was 50 ng/L.

The heart graft was initially transplanted into the first recipient (Recipient A), a 38‐year‐old man with blood group O Rh‐positive. The total ischemic time was 246 min. The immediate postoperative course was favorable, with extubation on postoperative day (POD) 1. The postoperative course was complicated by acute kidney injury, which resolved spontaneously. On POD 8, Recipient A developed a hemorrhagic stroke with a deep Sylvian hematoma that progressed to brain death on the same day. No donor‐specific antibodies directed against HLA‐A, HLA‐B, HLA‐DR, or HLA‐DQ antigens were detected in the first recipient.

Repeat cardiac assessment demonstrated preserved graft function, with a left ventricular ejection fraction of 63%, no regional wall motion abnormalities, and no requirement for inotropic or catecholaminergic support. Following the initial transplantation, the peak high‐sensitivity troponin concentration reached 1251 ng/L. Transthoracic echocardiography revealed an interventricular septal thickness of 9 mm. Assessment of right ventricular systolic function yielded discordant findings, with a preserved fractional area change (39%), but reduced tricuspid annular plane systolic excursion (7 mm) and tricuspid lateral annular systolic velocity (S ^′^ wave, 5 cm/s) in sinus rhythm. No tricuspid regurgitation was detected, and the right‐sided cardiac chambers were not dilated. As the first transplant and given the favorable posttransplant course, coronary angiography was not performed.

Reuse of this cardiac graft was subsequently proposed to our patient (Recipient B). ABO compatibility was maintained between the donor and both recipients; however, HLA mismatches were identified at the HLA‐A, HLA‐B, and HLA‐DR loci (Table 2).

**Table 2:** ABO compatibility and HLA mismatching.

	HLA‐A	HLA‐B	HLA‐DR	Group ABO
Donor	1/24	8/35	4/8	O+
First recipient	2/0	62/44	4/7	O+
Second recipient	2/30	38/57	4/7	O−

Orthotopic heart transplantation was performed using the bicaval anastomotic technique. The total ischemic time was 240 min. The graft recovered the spontaneous cardiac rhythm without electrical cardioversion. Cardiopulmonary bypass time was 132 min, including 99 min of aortic cross‐clamping and 31 min of circulatory assistance. The virtual crossmatch was negative for both the first and second donors. A physical crossmatch was subsequently performed with the second donor and was also negative. Donor‐specific antibodies were negative. The immediate postoperative course was complicated by difficult hemostasis requiring transfusion of 972 mL of fresh frozen plasma, 6 g of fibrinogen, and platelets as well as acute right ventricular failure necessitating inhaled nitric oxide at 20 ppm, isoproterenol (maximum dose 0.016 *μ*g/kg/min), and milrinone (maximum dose 0.69 *μ*g/kg/min). Shortly after admission to the intensive care unit (ICU), the patient developed severe vasoplegic shock refractory to high‐dose norepinephrine (up to 2.6 *μ*g/kg/min), in the absence of any septic etiology. The addition of arginine vasopressin at a dose of 1.6 IU/h resulted in the rapid resolution of vasoplegic syndrome.

The patient was extubated on POD 3. Subsequent ICU admission was favorable, allowing discontinuation of isoproterenol on POD 3, arginine vasopressin on POD 4, milrinone on POD 5, and norepinephrine on POD 9. He developed acute kidney injury, classified as KDIGO Stage 3, with a peak serum creatinine level of 255 *μ*mol/L, which evolved into chronic kidney disease with preserved urine output and without the need for renal replacement therapy. On POD 7, the patient presented with mild hypoxemia related to nosocomial pneumonia. Cytobacteriological examination of respiratory sputum identified wild‐type *Escherichia coli*, and intravenous ceftriaxone (2 g/day) was initiated for a 5‐day course. The patient was discharged from the ICU on POD 10. No additional blood product transfusions were required during hospitalization.

Immunosuppressive therapy consisted of intravenous tacrolimus with a target tacrolimus trough concentration between 13 and 20 *μ*g/L, achieved by Day 7, mycophenolate mofetil (1 g twice daily, intravenous), corticosteroids (1 mg/kg/day), and anti‐CD25 antibodies for induction (basiliximab, two intravenous doses of 20 mg on POD 1 and POD 4). During the subsequent hospital stay, the patient developed cavitary pneumonia caused by *Pseudomonas aeruginosa* associated with mild hypoxemia requiring standard oxygen therapy. The antibiogram demonstrated resistance to piperacillin–tazobactam and the presence of cephalosporinase, leading to a 4‐week course of meropenem and an 8‐week course of ciprofloxacin. The Aspergillus PCR test result was negative. Because the patient was discharged on POD 20, meropenem was administered intravenously via a PICC line at home to complete the full course treatment and ciprofloxacin was switched to oral administration. The follow‐up chest CT scan at 3 months showed complete healing of the cavitary pneumonia.

At 1‐month follow‐up, transthoracic echocardiography demonstrated preserved left ventricular systolic function and satisfactory right ventricular performance (tricuspid annular plane systolic excursion, 15 mm; S wave velocity, 10 cm/s), with no valvular abnormalities. An endomyocardial biopsy at 1 month revealed a limited number of dilated capillaries containing suspicious mononuclear inflammatory cells, without evidence of cellular rejection. Biopsies performed at 3 and 6 months demonstrated mild cellular rejection (grade 1R according to the ISHLT 2004–2013 working formulation) without antibody‐mediated rejection; these findings resolved spontaneously by 1 year without modification of the immunosuppressive therapy. At 1‐year follow‐up, the patient remained in excellent clinical condition, with preserved functional capacity (NYHA Class 1), and stable renal function (serum creatinine 1.43 mg/dL; estimated glomerular filtration rate (eGFR) 56 mL/min/1.73 m^2^). Transthoracic echocardiography showed preserved biventricular function. Immunological monitoring detected no donor‐specific antibodies at 1‐, 3‐ and 12‐months posttransplantation.

At 2‐ and 3‐year follow‐up, the patient continued to demonstrate excellent functional status (NYHA Class 1) and stable renal function, with serum creatinine level of 1.31 mg/dL and an eGFR of 57 mL/min. He reported an excellent quality of life as he resumed his sports and leisure activities and remained free of transplant‐related complications. Transthoracic echocardiography showed preserved left ventricular EF of 60%, no significant valvular abnormalities, and normal right ventricular size and function. Immunological assessment remained unremarkable, with no evidence of donor‐specific antibody development.

## 3. Discussion

We report a case of heart transplantation using a reused cardiac graft. Organ shortage remains a major limitation in increasing the number of heart transplantations worldwide, whereas the number of patients on the transplant waiting lists continues to rise. Therefore, expanding the donor pool has become a major priority in transplantation. Over the past few decades, several innovative strategies have been developed to address this challenge.

First of all, ex vivo normothermic or hypothermic perfusion systems have emerged as valuable tools in modern heart transplantation. These technologies allow long‐distance organ procurement, improve donor–recipient matching opportunities, and facilitate the use of extended‐criteria donors. Such donors may present with reduced left ventricular ejection fraction, left ventricular hypertrophy, history of cardiac arrest, prolonged ischemic time exceeding 4 h, or coronary artery disease. Furthermore, compared with traditional static cold storage, hypothermic ex vivo perfusion has been associated with a reduction in major adverse cardiac transplant events, suggesting a clinically meaningful benefit [1].

Furthermore, these devices have proven particularly valuable in heart transplantation programs using donations after circulatory death (DCD), because DCD protocols typically include a mandatory period following electromechanical arrest to confirm death, leading to a longer warm ischemic time.

For example, Schroder et al. evaluated the use of the Organ Care System (OCS) to resuscitate, preserve, and assess hearts from extended‐criteria donors. In their study, grafts were considered suitable for transplantation if arterial lactate levels remained < 5 mmol/L, and perfusion parameters were within the recommended ranges. Using these criteria, the authors reported a transplantation rate of 81% among donor hearts, with survival rates of 94.7% at 30 days and 88% at 6 months [2].

Secondly, domino heart transplantation procedure, in which a patient undergoing heart–lung transplantation donates their native heart to another recipient requiring an isolated heart transplant, has been used for several decades. In this situation, a patient undergoing heart–lung transplantation donates their native heart to another recipient requiring an isolated heart transplant. The domino hearts may have several advantages. In particular, the right ventricle is often well‐adapted to elevated pulmonary vascular resistance, which may be beneficial for certain recipients. In addition, the graft can be retrieved under controlled surgical conditions, and because the domino donor is a living donor, several adverse physiological changes typically associated with brain death can be avoided. These factors may contribute to improved early postoperative outcomes. Khaghani et al. reported survival rates of 75%, 70%, and 58% at 1, 5, and 10 years, respectively, among recipients of domino‐heart transplants [3].

Similarly, Maynes et al. described outcomes following domino heart transplantation, with a reported 1‐year survival rate of 86% and an overall mortality rate of 35% at a mean follow‐up of 37 months [4].

A third and far less common strategy is the reuse of previously transplanted cardiac grafts. Here, we describe the eight previous cases reported in the literature, as summarized in Table 3. The first cases were reported in the early 1990s, and they demonstrated encouraging outcomes. In 1993 and 1994, two initial reports described the successful retransplantation of a cardiac graft with good survival at 1 and 2 years, respectively [5, 6]. In one case, Pasic et al. reported that the second recipient experienced a moderate rejection episode that responded well to immunosuppressive therapy. Although ABO compatibility was maintained between the donor and the two recipients, HLA mismatching was present for the HLA‐A, HLA‐B, and HLA‐DR antigens [5].

**Table 3:** Previously reported cases of reused cardiac grafts.

Authors	Year	Interval between transplants	Ischemic time (minutes)	Rejection	Outcome/follow‐up	ABO compatibility	Immunosuppresion regimen
Pasic et al.	1993	13 days	Not specified	Moderate (Grade 2R)	Good recovery	O ‐‐> O ‐‐> B	Ciclosporine azathioprine corticosteroids antithymocyte globulin
Meiser et al.	1994	2 days	Not specified	Not specified	Alive at 2 years	Not specified	Not specified
Simsir et al.	2008	5 days	197	Minimal (Grade 1R)	Alive at 13 months	Not specified	Antithymocyte globulin, tacrolimus, mycophenolate mofetil, corticosteroids
Nemec et al.	2010	15 days	193	Minimal (Grade 1R)	Good recovery	O ‐‐> O+ ‐‐> O+	Anti‐CD5 antibodies, tacrolimus, mycophenolate mofetil, corticosteroids
Planinc et al.	2012	4 days	231	Minimal (Grade 1R)	Death at 10 months	Not specified	Not specified
Mullen et al.	2014	15 days	295	None	Alive at 15 months	Not specified	Not specified
Rodriguez‐Gonzalez et al.	2016	48 h	50	None	Alive at 4 months	A ‐‐ > A ‐‐ > A	AntiCD5 antibodies, ciclosporine, acid mycofenolic, corticosteroids
Esmailian et al.	2023	10 days	100	None	Alive at 7 months	O‐‐ > B ‐‐ > O	Antithymocyte globulin, tacrolimus, mycophenolate mofetil, corticosteroids
Present case	2022	8 days	240	Minimal (Grade 1R)	Alive at 3 years	O+ ‐‐> O+ ‐‐> O−	Anti‐CD5 antibodies, tacrolimus, mycofenolic acid and corticosteroids

Subsequently, several additional case reports confirmed the feasibility of this approach. Simsir et al. described the reuse of a cardiac graft 5 days after the first transplantation. The total ischemic time was 197 min for the retransplantation, and the postoperative course was uneventful, allowing discharge on POD 12 with preserved cardiac function confirmed by right‐heart catheterization. Endomyocardial biopsies revealed no or minimal (Grade 1R) cellular rejection under standard immunosuppressive therapy consisting of thymoglobulin induction, followed by tacrolimus, mycophenolate mofetil, and prednisone. The patient remained alive with excellent graft function 13 months after the surgery [7]. In 2010, another report described double transplantation using the same graft, with an interval of 15 days between the procedures. The total ischemic time was 193 min. Immunosuppressive therapy consisted of anti‐CD25 antibodies, tacrolimus, mycophenolic acid, and corticosteroids. The original donor had a blood group of O Rh−, and both recipients had O Rh+. The second recipient developed a Grade 1R rejection episode, which was successfully treated with increased corticosteroid therapy. All subsequent biopsies showed no evidence of rejection, and the patient remained in excellent condition for more than 10 months after transplantation [8].

Planinc et al. reported a similar case involving retransplantation 4 days after the initial procedure. Despite a prolonged ischemic time of 231 min, the recipient experienced satisfactory postoperative recovery and was discharged on POD 35, with preserved cardiac function. Results from the first cardiac biopsy showed 1R cellular rejection. Ten months after transplantation, the patient died of acute myelogenous leukemia unrelated to graft function [9]. Mullen et al. also reported successful retransplantation 15 days after the initial transplant. Despite a prolonged ischemic time of 295 min and transient right ventricular dysfunction, the patient recovered well and was discharged on POD 22, remaining alive and clinically stable 15 months after transplantation. No episodes of rejection were observed using the standard triple‐therapy immunosuppression regimen. [10]

Rodriguez‐Gonzalez et al. described a case of graft reuse 48 h after the initial transplantation, with a particularly short ischemic time of only 50 min. The donors and recipients shared blood group A. The postoperative course was favorable, with no significant acute rejection observed on surveillance biopsies under a standard immunosuppressive regimen, including methylprednisolone, basiliximab, cyclosporine, mycophenolate mofetil, and prednisone. The patient remained asymptomatic 4 months after transplantation [11].

More recently, in 2023, Esmailian et al. reported a case of cardiac graft reuse 10 days after initial transplantation. The ischemic time was 188 min for the first transplant and 100 min for the second. Immunosuppressive therapy consisted of antithymocyte globulin induction, followed by cyclosporine, mycophenolate mofetil, and corticosteroids. Follow‐up showed excellent cardiac function, with the recipient remaining alive and clinically stable 7 months after transplantation. There was little concern regarding hyperacute rejection because the original donor heart was transplanted into the first recipient with B Rh‐positive blood. When the original O Rh‐positive donor heart was reallocated to the second recipient with O Rh‐positive blood, there was some concern that the type B blood from the first recipient could be carried over and may result in hemolysis. However, there is no clinical or laboratory evidence of hemolysis. Immunosuppressive therapy consisted of antithymocyte globulin induction, followed by tacrolimus, mycophenolate mofetil, and corticosteroids. No evidence of rejection was found on any transplant endomyocardial biopsy. Follow‐up showed excellent cardiac function, with the recipient remaining alive and clinically stable 7 months after transplantation [12].

This report describes the ninth case of cardiac graft reuse worldwide, which was first reported in France. A 61‐year‐old man was admitted to the intensive cardiac care unit with cardiogenic shock secondary to dilated cardiomyopathy related to atrial fibrillation. Despite maximal medical therapy, the patient remained hemodynamically unstable and dependent on inotropic support, prompting an urgent consideration for heart transplantation. Examination of the donor heart showed preserved left ventricular ejection fraction, absence of segmental wall motion abnormalities, in the absence of inotropic support in the donor. The assessment of right ventricular function yielded discordant findings. Although parameters reflecting longitudinal right ventricular systolic function were reduced, these alterations were not considered indicative, as such indices are frequently impaired following cardiopulmonary bypass and may not accurately reflect intrinsic right ventricular performance in the early postoperative period [13, 14]. Moreover, right ventricular fractional area change remained preserved and has been reported to provide a more reliable echocardiographic assessment of right ventricular systolic function after cardiopulmonary by‐pass [15]. Coronary angiography was not performed before either transplantation. The patient′s risk of atherosclerotic was considered low based on his young age, occasional cannabis use and limited history of tobacco exposure. In addition, given the favorable improvement in graft function following transplantation, the clinician team elected to defer invasive coronary evaluation.

The organ was transported using standard static cold storage with a cold ischemic time of 240 min. Although ex vivo perfusion would have allowed further functional assessment of the graft before implantation, this technology was not available at our center at the time of transplantation. Similar to several previously reported cases, the total ischemic time exceeded 180 min. Despite this prolonged ischemic period and the theoretical risk of ischemia‐reperfusion injury, particularly affecting the right ventricular function, the postoperative course was favorable. The patient demonstrated rapid recovery of cardiac function without evidence of severe primary graft dysfunction or refractory right ventricular failure.

In previously reported cases, postoperative outcomes were generally favorable, with early hospital discharge and preserved graft function. In our case, the patient developed transient vasoplegic shock that resolved within 48 h. Such hemodynamic instability is relatively common after heart transplantation and is not specific to reused cardiac grafts.

Immunological considerations remain a central concern in graft reuse. The allograft is exposed to the immune systems of two different hosts, theoretically increasing the risk of immune sensitization in the second recipient. Conversely, some authors hypothesized that prior exposure of the graft to immunosuppressive therapy in the first recipient may reduce immunogenicity and potentially lower the risk of rejection. Interestingly, in most published cases, surveillance biopsies demonstrated either no rejection or only mild cellular rejection episodes, which responded well to standard immunosuppressive therapy. In most reports, immunosuppressive regimens consisted of induction therapy with antithymocyte globulin or anti‐CD25 antibodies, followed by maintenance therapy with corticosteroids, mycophenolate mofetil, and either tacrolimus or cyclosporine [5–8, 11]. In our case, patients were of blood O group; however, the second recipient was Rh‐negative. No evidence of hemolysis was observed during hospitalization. HLA analysis revealed substantial mismatching, including two HLA‐A, two HLA‐B, and one HLA‐DR mismatches (Table 2) for donor and second recipient. No donor‐specific antibodies were detected throughout the 3‐year follow‐up period. Furthermore, episodes of mild acute cellular rejection identified at 3‐ and 6‐months posttransplantation resolved favorably.

However, only a limited number of cases have been reported to date, and the safety and long‐term outcomes of reused cardiac grafts remain poorly documented. In most published reports, the follow‐up duration did not exceed 12–18 months. In contrast, our patient remained in good clinical condition with preserved graft function and satisfactory functional capacity at 3‐year follow‐up. Longer follow‐up periods would be valuable for better assessment of long‐term graft performance and patient quality of life. Nevertheless, given the rarity of such procedures, large‐cohort studies are unlikely to be feasible.

From a surgical standpoint, all retransplantations were performed within the first 15 PODs. No technical difficulties related to redo sternotomy were reported, and all procedures were completed uneventfully. The feasibility and risks of later retransplantation should nevertheless be discussed, particularly in view of the higher risk of re‐entry injury.

Finally, ethical considerations must be addressed. Nakazawa et al. analyzed the legal and ethical aspects of organ reuse in the United States and Japan and concluded that this practice is not illegal and may be ethically acceptable in the context of severe organ shortages [16]. In our case, the French National Organ Procurement Organization Agence de la Biomédecine gave her authorization for graft reuse. Considering the exceptional nature of cardiac allograft reuse, the decision of retransplantation was reviewed by the heart transplantation team of our institution from a medical standpoint. Consent for organ donation was obtained from the family of the first recipient.

In conclusion, we report the first case of cardiac graft reuse performed in France and the ninth reported case worldwide. Cardiac graft reuse may represent, after a thorough assessment and a multidisciplinary decision, a potential strategy in carefully selected situations. Additional reports and longer follow‐up data are needed to better evaluate the long‐term graft function, recipient outcomes, and quality of life following this highly uncommon procedure.

**Nomenclature**
DBD: donation after brain death
DCD: donation after cardiac death
ECMO: extracorporeal membrane oxygenation
EF: ejection fraction
HLA: Human leucocyte antigen
ICU: intensive care unit
ISHLT: international society of heart and lung transplantation
PCR: polymerase chain reaction
POD: postoperative day

## Author Contributions

Alexis Krin wrote the case report. Jean Porterie and Myriam Addi contributed to the conception of this case.

## Funding

No funding was received for this manuscript.

## Consent

Patient gave oral and written consent for the publication of the case report.

## Conflicts of Interest

The authors declare no conflicts of interest.

## Data Availability

The data that support the findings of this study are available from the corresponding author upon reasonable request.
